# Supplementary figures and images for: Primary care interventions to address physical frailty among community-dwelling adults aged 60 years or older: A meta-analysis
Source: PLoS One. 2020 Feb 7;15(2):e0228821. doi: 10.1371/journal.pone.0228821 (PMC7006935; doi:10.1371/journal.pone.0228821)

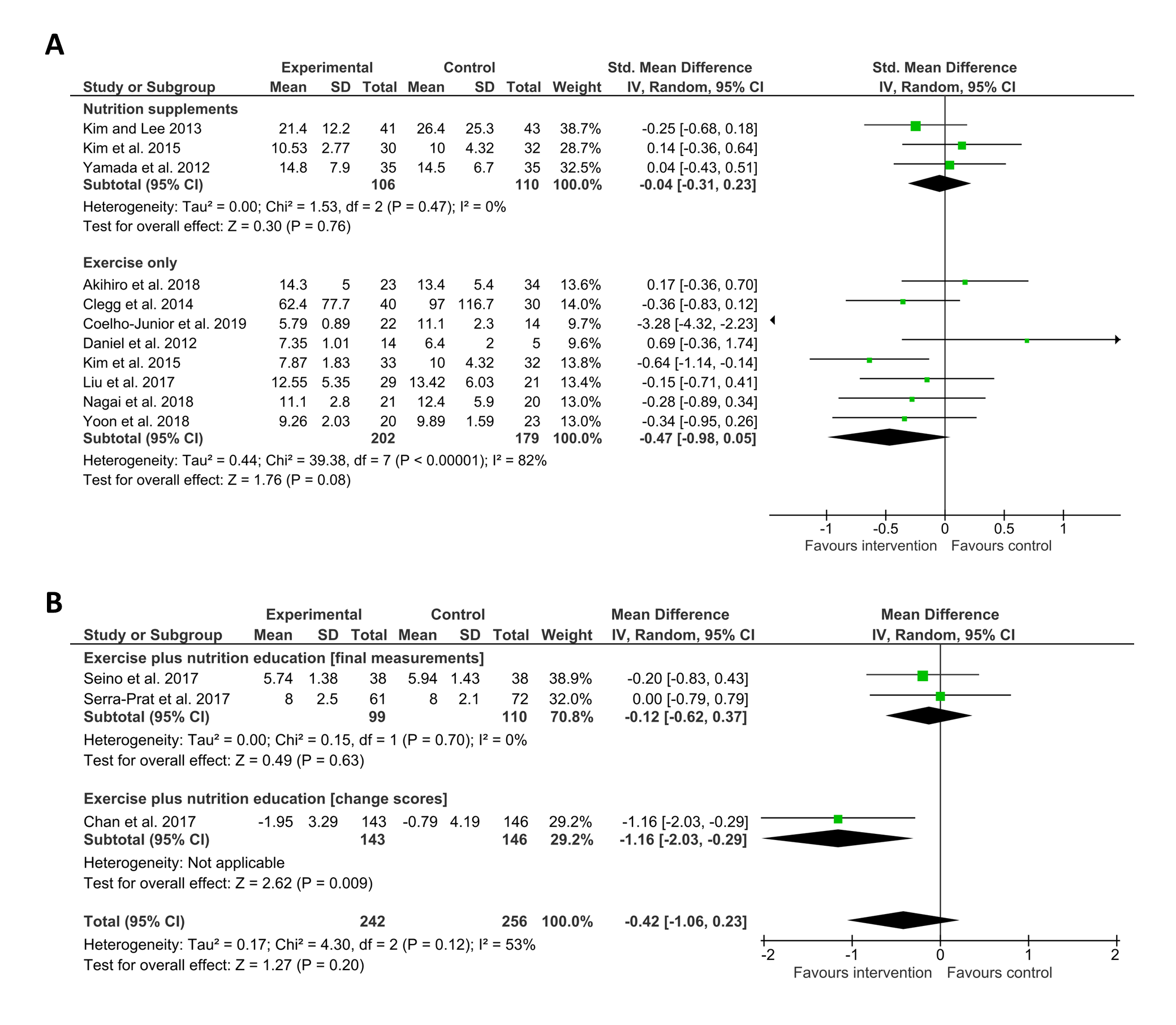

Supplement: S1 Fig — A) Shows SMD (square data markers) with 95% CI (horizontal lines) for meta-analysis of mean ± SD time taken to perform timed up and go test (measured in seconds), for interventions using nutrition supplements or exercise alone. B) Shows Mean Difference with 95% CI for meta-analysis of time taken to perform TUG (measured in seconds), reported either as change scores or as final measurements ± SD, separated into sub-groups for clarity, for interventions using exercise plus nutrition education. Size of data markers indicates study weighting in random effects meta-analysis. Diamond-shaped data markers indicate overall SMD or Mean difference with CI for each grouping of interventions. I2 statistic is reported for each group. Left side favours intervention. (TIF) [file pone.0228821.s004.tif]

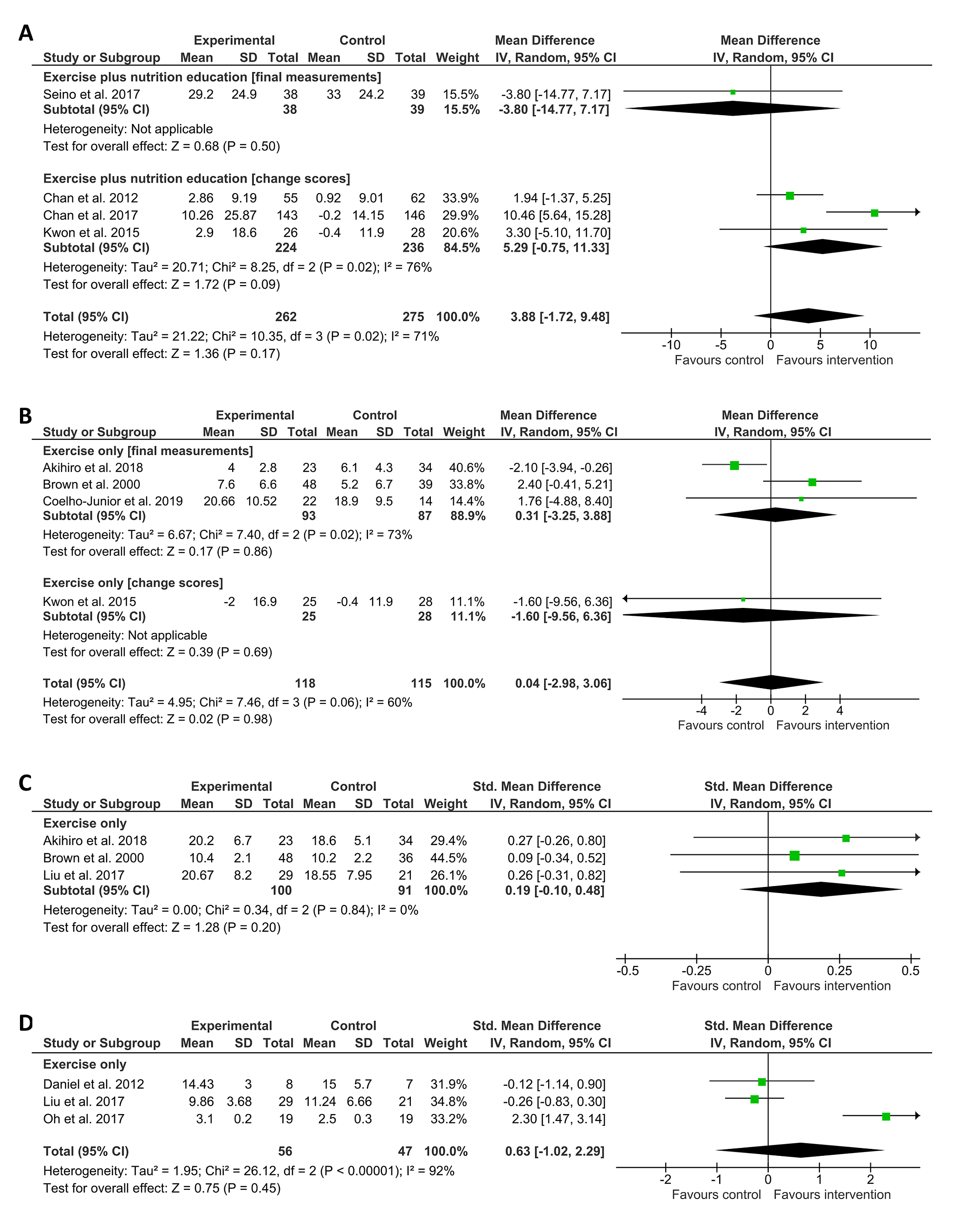

Supplement: S2 Fig — A, B) Show Mean Difference (square data markers) with 95% CI (horizontal lines) for meta-analysis of single-leg balance (measured in seconds) reported either as change scores or as final measurements ± SD, separated into sub-groups for clarity, for interventions using exercise plus nutrition education and exercise only. C) Shows SMD with 95% CI for meta-analysis of mean scores (measured in centimetres or inches) for functional reach ± SD, for interventions using exercise. D) Shows SMD with 95% CI for meta-analysis of mean scores in chair stand test (measured as number of stands in 30 seconds or time taken to rise 5 times from sitting) ± SD for interventions using exercise. Size of data markers indicates study weighting in random effects meta-analysis. Diamond-shaped data markers indicate overall SMD or Mean difference with CI for each grouping of interventions. I2 statistic is reported for each group. Right side favours intervention. (TIF) [file pone.0228821.s005.tif]

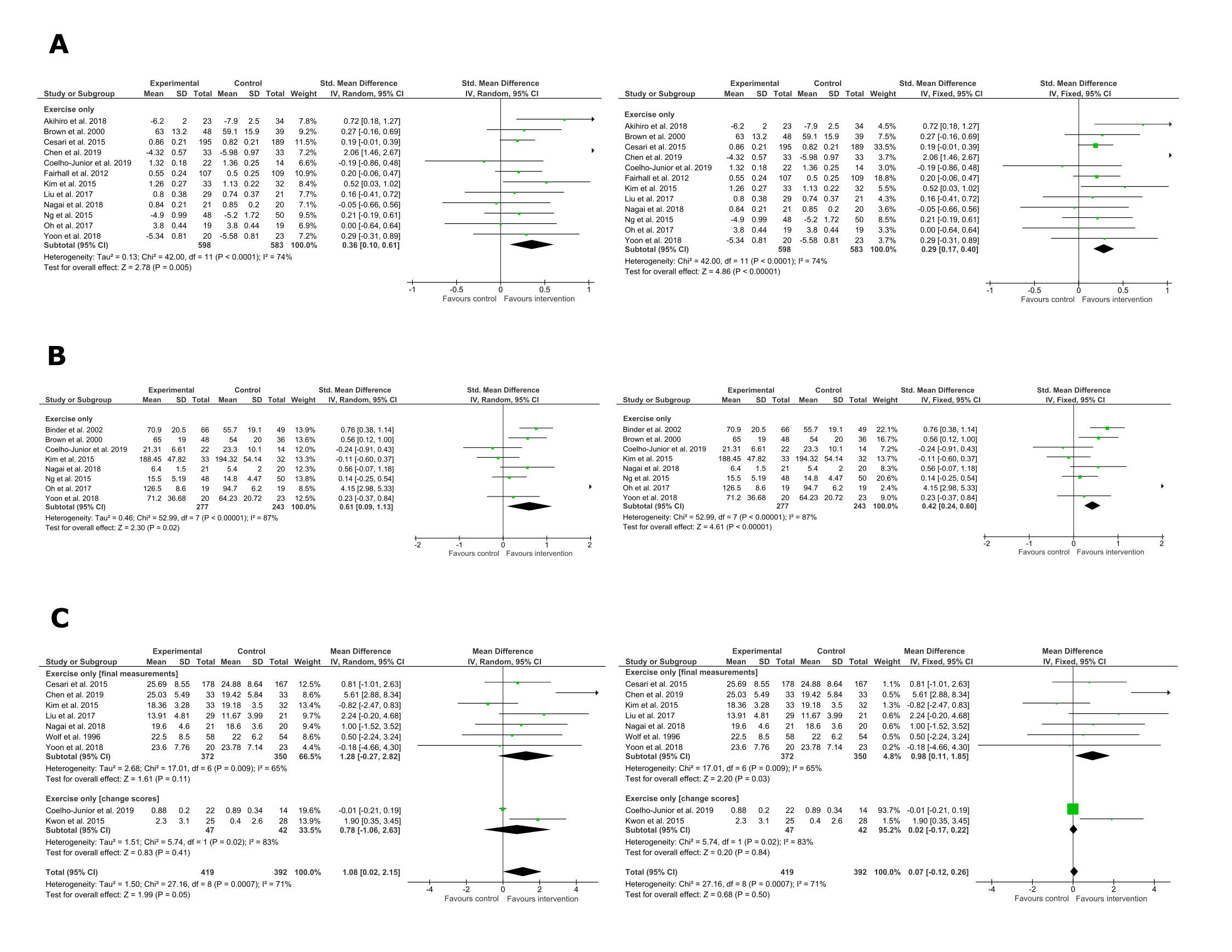

Supplement: S3 Fig — Left side shows meta-analysis using Random Effects model; right side shows meta-analysis using Fixed Effects model. A) Shows effect (SMD) of exercise interventions on gait speed, B) shows effect (SMD) of interventions on leg strength, and C) shows effect (Mean Difference) of interventions on grip strength. Size of data markers indicates study weighting in meta-analysis. Diamond-shaped data markers indicate overall SMD or Mean difference with CI for each grouping of interventions. I2 statistic is reported for each group. Right side favours intervention. (TIF) [file pone.0228821.s006.tif]

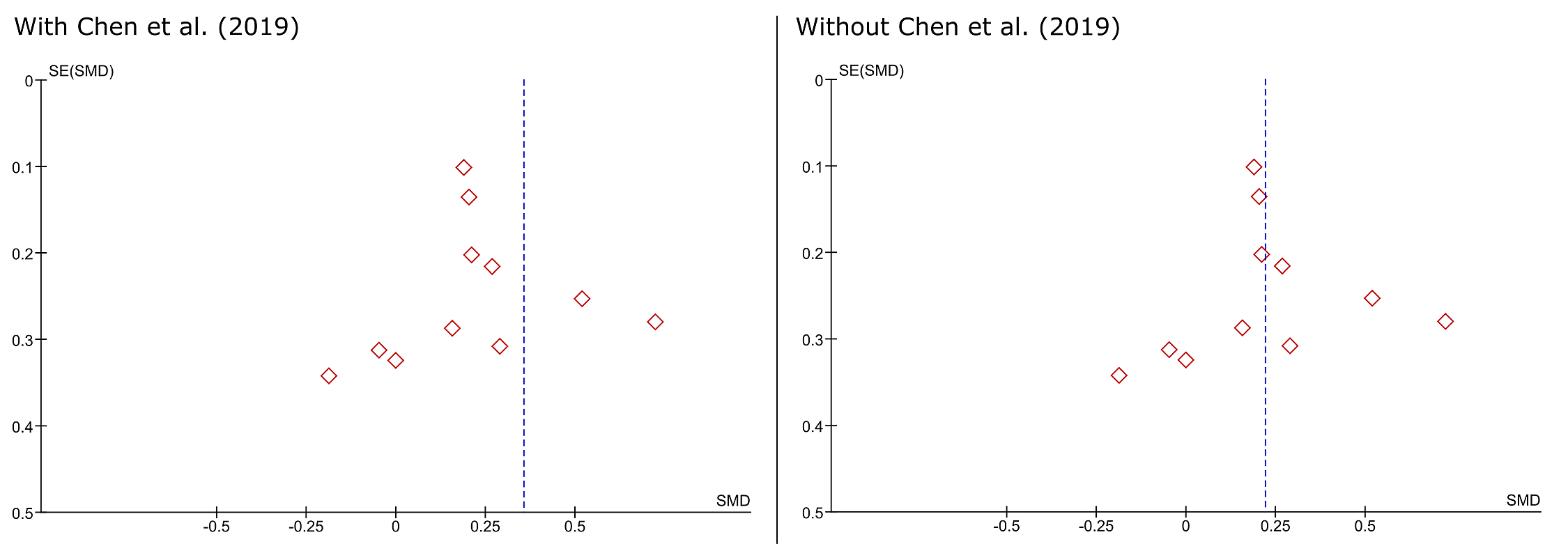

Supplement: S4 Fig — Open diamond shapes represent individual studies in the meta-analysis. Y axis shows SE of the SMD; X axis shows SMD. Blue dashed vertical line represents overall effect. Left panel shows funnel plot including the study by Chen et al. (2019); right panel shows funnel plot excluding Chen et al. (TIF) [file pone.0228821.s007.tif]
