# Supplementary material for: Primary care interventions to address physical frailty among community-dwelling adults aged 60 years or older: A meta-analysis
Source: PLoS One. 2020 Feb 7;15(2):e0228821. doi: 10.1371/journal.pone.0228821 (PMC7006935; doi:10.1371/journal.pone.0228821)
Supplement: Attachment — Submitted filename: Response to reviewers.docx [file pone.0228821.s008.docx]

**15 November 2019**

Dear review team,

We express our thanks for getting our manuscript reviewed so quickly and for sending on comments. We now submit a revised manuscript which includes the requested changes. These changes are easily seen in the version with track changes, and we have also uploaded a clean version with no tracking.

Specifically:

**1)** We have updated the formatting style to conform to PLOS guidelines, and renamed supporting material files accordingly.

**2)** We have updated the PRISMA checklist to address all items, and we confirm that the study is labelled as a meta-analysis in the title, “**Primary care interventions to address physical frailty among community-dwelling adults aged 60 years or older: A meta-analysis.”**

**3)** We have addressed the reviewer’s comment, *“As a proposal for improvement, I suggest that the time frame of the publications selected for analysis be included in the methodology section, between lines 94 and 139,”* by adding a sentence to the methodology section, which can be seen in the revised manuscript, commencing at line 100.

Many thanks for your time and attention.

With kind regards,

**Stephen H-F. Macdonald PhD.**

Research Scientist

Health Systems Team

School of Nursing, Midwifery, and Health Systems

University College Dublin

Belfield

Dublin D4

Ireland

+353 (0)86 211 2436

[macdonaldez@gmail.com](mailto:macdonaldez@gmail.com)

With **John Travers, Éidín Ní Shé, Jade Bailey, Roman Romero-Ortuno, Michael Keyes, Diarmuid O’Shea,** and **Marie Therese Cooney**
